# Constrictive pleuropericarditis: a dominant clinical manifestation in Whipple’s disease

**DOI:** 10.1186/1471-2334-13-579

**Published:** 2013-12-09

**Authors:** George Stojan, Michael T Melia, Sandeep J Khandhar, Peter Illei, Alan N Baer

**Affiliations:** 1 Department of Medicine, Harvard Medical School, Boston, MA, USA; 2 Department of Medicine, Division of Infectious Diseases, Johns Hopkins University School of Medicine, Baltimore, MD, USA; 3 Division of Thoracic Surgery, Cardiac, Vascular & Thoracic Surgery Associates, P.C. and Inova Fairfax Hospital, Falls Church, VA, USA; 4 Department of Pathology, Johns Hopkins University School of Medicine, Baltimore, MD, USA; 5 Department of Medicine, Division of Rheumatology, Johns Hopkins University School of Medicine, Baltimore, MD, USA

**Keywords:** Whipple’s disease, *Tropheryma whipplei*, Constrictive pericarditis, Pleuritis

## Abstract

**Background:**

Whipple’s disease is a rare, multisystemic, chronic infectious disease which classically presents as a wasting illness characterized by polyarthralgia, diarrhea, fever, and lymphadenopathy. Pleuropericardial involvement is a common pathologic finding in patients with Whipple’s disease, but rarely causes clinical symptoms. We report the first case of severe fibrosing pleuropericarditis necessitating pleural decortication in a patient with Whipple’s disease.

**Case presentation:**

Our patient, an elderly gentleman, had a chronic inflammatory illness dominated by constrictive pericarditis and later severe fibrosing pleuritis associated with a mildly elevated serum IgG4 level. A pericardial biopsy showed dense fibrosis without IgG4 plasmacytic infiltration. The patient received immunosuppressive therapy for possible IgG4-related disease. His poor response to this therapy prompted a re-examination of the diagnosis, including a request for the pericardial biopsy tissue to be stained for *Tropheryma whipplei*.

**Conclusions:**

Despite a high prevalence of pleuropericardial involvement in Whipple’s disease, constrictive pleuropericarditis is rare, particularly as the dominant disease manifestation. The diagnosis of Whipple’s disease is often delayed in such atypical presentations since the etiologic agent, *Tropheryma whipplei,* is not routinely sought in histopathology specimens of pleura or pericardium. A diagnosis of Whipple’s disease should be considered in middle-aged or elderly men with polyarthralgia and constrictive pericarditis, even in the absence of gastrointestinal symptoms. Although *Tropheryma whipplei* PCR has limited sensitivity and specificity, especially in the analysis of peripheral blood samples, it may have diagnostic value in inflammatory disorders of uncertain etiology, including cases of polyserositis. The optimal approach to managing constrictive pericarditis in patients with Whipple’s disease is uncertain, but limited clinical experience suggests that a combination of pericardiectomy and antibiotic therapy is of benefit.

## Background

Whipple’s disease is a rare, multisystem, chronic infectious disease caused by the rod-shaped bacillus, *Tropheryma whipplei*. It preferentially affects middle-aged Caucasian men of European descent. The classic presentation is marked by chronic diarrhea with arthralgia, abdominal pain, weight loss and malabsorption. However, some patients may present with symptoms referable to other organ systems, thereby delaying the diagnosis.

Serosal inflammation is a common finding in post-mortem studies of patients with Whipple’s disease. The most commonly involved serosal surfaces are the pericardium and pleura. In the original patient reported by George Whipple in 1907, the pericardial cavity was entirely replaced with loose edematous granulation tissue [1]. Pericardial adhesions were noted in 26-79%, while fibrous pleuritis was evident in 30-78% of post-mortem examinations of patients with Whipple’s disease [2-5]. Pleural and pericardial abnormalities frequently co-exist in Whipple’s disease [6]. Surprisingly, pericarditis is a rare clinical finding in patients with Whipple’s disease, either because the manifestations remain subclinical or the symptoms are overshadowed by more prominent ones stemming from other organ systems. There is no direct correlation between the severity and duration of the systemic disease and the extent of cardiac involvement [2]. There are only two previously reported cases in which constrictive pericarditis was the primary or sole presentation of Whipple’s disease [7,8] and a total of six previously reported cases of constrictive pericarditis, all affecting middle aged men [7-12] (Table 1). The time between onset of symptoms and pericardial involvement ranged from a few months to several decades. Two patients who had histologically-proven pericardial involvement had normal small bowel biopsies [9,11]. Four of the six patients had pleural effusions that did not require further intervention [8-11]. All patients underwent pericardiectomy and received antibiotic therapy with clinical improvement.

**Table 1:** Previously reported cases of constrictive pericarditis in Whipple’s disease

**Study**	**Age at Diagnosis, Sex**	**Prodromal symptoms**	**Delay between prodromal stage and pericardial involvement**	**Pericardial histologic results**	**GI Histologic results**	**Pleural effusions**	**Treatment**	**Outcome**
Vlietstra RE et al. [12]	63, male	Arthralgia, Weight loss	8 years	PAS + histiocytes, electron microscopy+	Duodenal: PAS+, electron microscopy+	N/A	Tetracycline, pericardiectomy	
Crake et al. [7]	37, male	Chest pain, shortness of breath	0	PAS + macropages	Duodenal: PAS+	N/A	Pericardiectomy, tetracycline	Survived
Freychet et al. [11]	61, male	Arthralgia, diarrhea	Not specified	PAS + macrophages; electron microscopy +	Jejunal: negative	Unilateral	N/A	N/A
Iqbal et al. [8]	58, male	Orthopnea, lymphadenopathy, ascites	0	PAS + macrophages; electron microscopy +	Jejunal: PAS + histiocytes	Unilateral, transudate	Ceftriaxone, tetracycline, pericardiectomy	Survived
Makol et al. [9]	56, male	Seronegative inflammatory arthritis	20 years	PAS + macrophages; *T.whipplei* PCR+	Jejunal: negative	Bilateral	Ceftriaxone, Bactrim, pericardiectomy	Survived
Sutherland et al. [10]	45, male	Anemia, weight loss, lymphadenopathy	6 months	N/A	Duodenal: PAS+, electron microscopy+	bilateral	Ceftriaxone, Bactrim, pericardiectomy	Survived

Whipple’s disease may be associated with prominent pulmonary manifestations producing a symptom complex of dyspnea, pleuritic chest pain, and cough, reflecting underlying parenchymal involvement or pleural effusions and thickening. Pleural effusions are present in approximately 10% of patients at the time of diagnosis [13]. Fibrosing pleuritis may be manifested in Whipple’s disease by pleural adhesions (identified by radiographs or at post-mortem examination) and by progressive reduction in lung volumes (shrinking lung syndrome) [14]. *Tropheryma whipplei* may be identified in the pleural fluid by PCR [15,16] or by pleural biopsy. No cases requiring pleural decortication have been described in the literature, although clinically significant loculated pleural effusions and pleural adhesions have been described [17,18].

## Case report

The patient, a retired mechanical engineer, was 68 years of age at the time of presentation to our clinic. He had a three-year history of migratory joint and muscle pain, affecting the feet, legs, shoulders, wrists and hands, and neck. Each episode of pain averaged 2–3 days and was occasionally associated with swelling, especially in the feet. Seven months prior to his initial evaluation in our clinic, he noted a productive cough and then, one month later, progressive lower extremity swelling with a 30-pound weight gain. He denied fever, abdominal pain, diarrhea, or anorexia. His past medical history was notable for hypertension. On examination, he was afebrile with a blood pressure of 160/95 mm Hg and weight of 194 pounds. There was pitting edema of both lower extremities to the level of the pelvis, with involvement of the scrotum and penile shaft. There was a small right axillary node. There was jugular venous distention to midneck while supine and decreased breath sounds at the lung bases. The heart sounds were normal without a pericardial knock.

The hemoglobin was 8.6 g/dl, WBC 8000/mm^3^, and platelets 521,000/mm^3^. ESR was 98 mm/hr and CRP 4.7 mg/dl. Urinalysis was normal without protein. Creatinine was 0.8 mg/dl, albumin was 3.2 g/dl, and serum aminotransferases were normal. Tests for antinuclear, extractable nuclear antigen, cyclic citrullinated peptide and neutrophil cytoplasmic antibodies were negative. Rheumatoid factor was 69 IU/ml. Ferritin was 60 ng/ml. Immunoglobulin G level was 2390 mg/dl. Serum protein electrophoresis showed a polyclonal hypergammaglobulinemia.

During an earlier evaluation, a bone marrow examination had shown active hematopoiesis, myeloid hyperplasia, and subtle myelofibrosis. The myeloid maturation pattern was atypical. Cytogenetic analysis was normal and tests for the JAK2 and BCR/ABL mutations were negative.

The patient was treated with diuretics with significant improvement in his anasarca. An extensive diagnostic evaluation was completed over the next 3 months. A transthoracic echocardiogram showed a restrictive filling pattern with an ejection fraction of 60-65%. On computed tomography (CT) scans of the chest, abdomen and pelvis, there were bilateral pleural effusions but no ascites. Small retroperitoneal nodes were noted. The pleural fluid was transudative. No abnormal fluorodeoxyglucose uptake was noted on a positron emission tomography/CT scan. An abdominal fat pad aspirate did not show amyloid. Celiac disease serologic tests were negative and stool alpha-1 antitrypsin levels were normal. An IgG4 level was 170 mg/dl. An evaluation of visual floaters showed dot and blot retinal hemorrhages.

Magnetic resonance imaging of the heart showed a mildly thickened pericardium with focal adhesions to the myocardium. The inferior vena cava was dilated, suggestive of constrictive pericarditis. On cardiac catheterization, the right and left heart filling pressures were increased and there was an early rapid diastolic filling pattern with equalization of the diastolic pressures.

**Figure 1:**
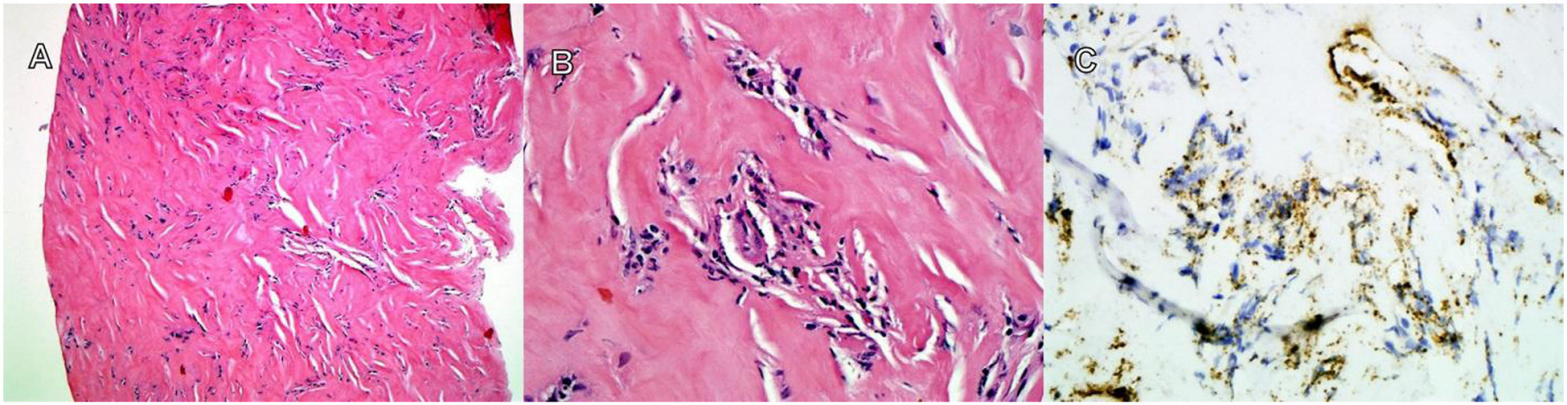
**Pericardial biopsy.** There is dense hyalinized fibroconnective tissue **(panel A**, 40 ×, hematoxylin-eosin stain**)** with focal minimal chronic inflammation **(panel B**, 400 ×, hematoxylin-eosin**)**. Immunostaining with antibodies to *Tropheryma whipplei* was positive in the cellular infiltrates **(panel C**, 400 ×, *Tropheryma whipplei* immunostain**)**.

Four months following the initial presentation, the patient underwent a video-assisted pericardial biopsy. The pericardium consisted of dense hyalinized fibrocellular tissue with focal areas of minimal chronic inflammation (Figure 1). Stains for amyloid and IgG4 plasma cells, prompted by the clinical findings, were negative. Pericardial fluid was not sampled during this procedure.

The patient was treated empirically for possible IgG4-related disease with prednisone 40 mg daily and noted improvement in his exertional dyspnea. His pleural effusions improved. However, his erythrocyte sedimentation rate and C-reactive protein levels remained elevated. Mycophenolate mofetil, 2 grams daily, was added two months later. The prednisone was tapered to a maintenance dose of 7.5 mg daily. Over the ensuing year, the patient continued to lose weight. Azathioprine was substituted for mycophenolate. The patient tolerated this medication poorly and lost weight loss rapidly. His serum albumin fell. He was readmitted when he presented with worsening dyspnea due to an enlarging pleural effusion. The original diagnosis was reconsidered due to the patient’s poor response to immunosuppressive therapy.

**Figure 2:**
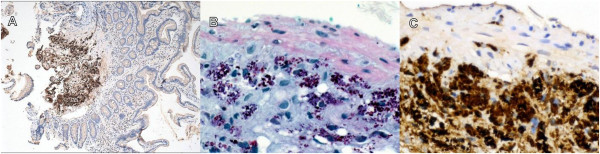
**Small bowel biopsy.** There is minimal histiocytic infiltration of the lamina propria **(panel A**, 40 ×, *Tropheryma whipplei* immunostain with hematoxylin counterstain). Macrophages that stain with periodic acid-Schiff **(panel B**, 400 ×**)** and *Tropheryma whipplei* immunostain **(panel C**, 400 ×**)** are densely aggregated in the submucosa, an unusual location in Whipple’s disease.

With Whipple’s disease entertained as a possible explanation for the pericardial disease, a request was made to re-examine the original pericardial biopsy. Immunostains for CD68 and *Tropheryma whipplei* were both positive in the same cells. Multiple small bowel biopsies obtained via enteroscopy showed numerous periodic acid-Schiff (PAS)-positive macrophages in the submucosa (Figure 2). Immunostains for *Tropheryma whipplei* were positive. Due to mild ataxia and leg stiffness, a cerebrospinal fluid analysis was performed, was normal in terms of cell count and chemistry, but positive for *Tropheryma whipplei* by polymerase chain reaction (PCR).

The patient was treated with intravenous ceftriaxone 2 grams daily for 2 weeks. He was then maintained on trimethoprim-sulfamethoxazole 800/160 mg bid, doxycycline 100 mg bid and hydroxychloroquine 200 mg tid. Low-dose prednisone was continued for the first six weeks of his antibiotic therapy to prevent immune reconstitution syndrome.

**Figure 3:**
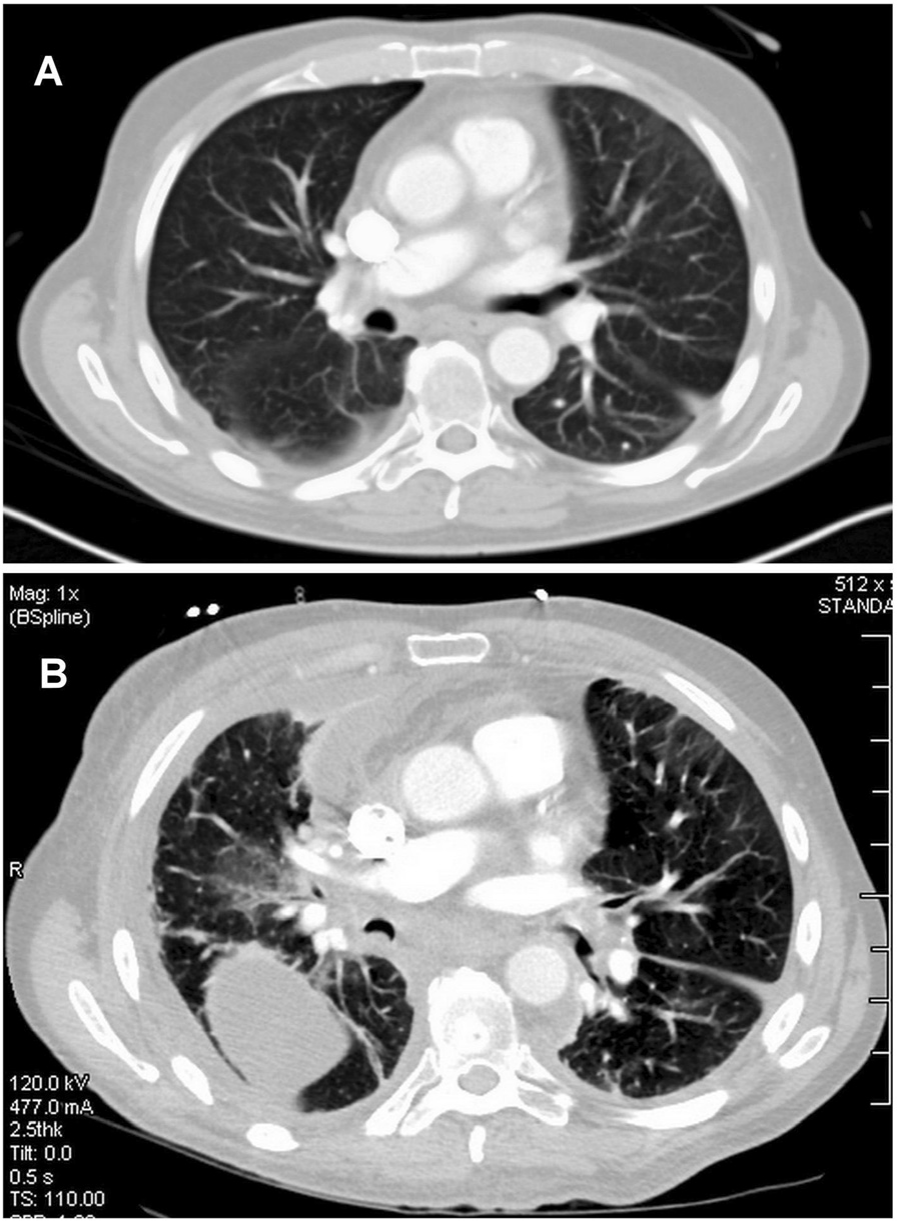
**Computed tomography of the chest.** Equivalent axial tomographic sections from November 2011 **(panel A)** and from December 2012 **(panel B)** are shown. Pericardial thickening measuring up to 6 mm is seen in the first tomogram. The interval development of circumferential pleural thickening and enlargement of the mediastinal shadow is evident in the second tomogram. The changes are more pronounced in the right pleural space, where there is a loculated effusion. Differences in the diameter of the large airways between the two tomograms may relate to differences in the respiratory cycle.

Three months after the initiation of antibiotics, the patient developed worsening pain in his right scapula that arose with ambulating or standing. This was ultimately associated with severe dyspnea. The patient was admitted to a local hospital. He was found to have a trapped right lung (Figure 3). A pleural fluid sample was bloody. At video-assisted thoracoscopic surgery, there was a dense, thick, visceral pleural rind and multiloculated gelatinous and serous pleural fluid collections. A complete decortication was performed. The pleura showed chronic inflammation, cellular fibrosis, and foci rich in interstitial macrophages containing intracytoplasmic PAS-positive bodies. An immunostain confirmed the presence of *Tropheryma whipplei*.

While he had resolution of his shoulder pain following his thoracic surgery, the patient continued to lose weight and died suddenly eight weeks following the pleural decortication. There was no post-mortem examination.

### Discussion

Our patient had a clinical syndrome of constrictive pleuropericarditis, an entity which has been reported rarely, but not in Whipple’s disease. The first published report dates back to 1941 where it was described in a case of disseminated tuberculosis [19]. Constrictive pleuropericarditis was also described in a case of thoracic crush injury [20] and in a young male with coarctation of the aorta and hepatic carcinoma [21]. The most recent published report described chronic pleuropericarditis complicated by lung entrapment and constrictive pericarditis in the setting of IgG4-related systemic disease [22].

To our knowledge, our patient was the second in whom *Tropheryma whipplei* was identified in pleural tissue by histological analysis in a biopsy specimen [23] and only the seventh with constrictive pericarditis reported in the world’s literature (Table 1). Importantly, the patient’s pleural effusions were transudative at the time of his initial evaluation and became hemorrhagic by the time of his pleural decortication. The utility of pleural fluid analysis for the detection of Whipple’s related pleural disease cannot be assessed from our experience with this patient.

The current diagnostic algorithm for Whipple’s disease mandates two out of three tests (PAS staining, PCR, or immunohistochemistry) be positive to assure a diagnosis; only a tentative diagnosis can be made with a single positive test [24]. Upper gastrointestinal endoscopy of the small intestine is still the first diagnostic test of choice [25]. The histology of small bowel biopsies is characterized by foamy, PAS-positive, diastase-resistant macrophages in the lamina propria. PAS staining alone is not completely specific. PAS-positive macrophages are also found in patients with infections caused by *Mycobacterium avium-intracellulare*, *Rhodococcus equi*, *Bacillus cereus*, *Corynebacterium* spp., *Histoplasma capsulatum*, or other fungi. Some of the histopathological features of Whipple’s disease are known also to occur in melanosis coli, histiocytosis, Crohn’s disease, and Waldenström’s macroglobulinemia [26,27]. In cases in which gastrointestinal symptoms are absent and the small bowel PAS staining is negative, biopsies from other affected organs should be obtained. PAS-positive cells have been described in a variety of tissues depending on the clinical presentation: in the cerebrospinal fluid and brain biopsy samples, lymph nodes, synovial fluid and tissue, cardiac valves, bone marrow and others [24].

PCR is increasingly used in the diagnosis of Whipple’s disease, but clinicians need to be aware of several different factors influencing its test performance. Native clinical specimens give more accurate results when compared to formalin-fixed tissue due to partial degradation of the DNA [28]. Suitable specimens for PCR analysis are duodenal tissue, heart valves, lymph nodes, stool, vitreous humor, and synovial or cerebrospinal fluid [27]. PCR may also be positive in samples from clinically affected sites in Whipple’s disease, including pleural fluid or pleural tissue as in the present case [27-29]. In a recent study of patients with culture-negative endocarditis, *Tropheryma whipplei* was the fourth most common cause of endocarditis, with the diagnosis being established through histologic, microbiologic and molecular analyses of explanted valves [30]. The detection of *Tropheryma whipplei* DNA in peripheral blood samples of patients with proven disease is often not positive [28,31]. Additionally, a positive peripheral blood PCR result does not confirm the diagnosis since false positive results may arise due to the presence of *Tropheryma whipplei* in healthy carriers [32,33]. Immunohistochemistry tests using specific antibodies against *Tropheryma whipplei* are more sensitive than PAS staining, and the organism can be identified by immunohistochemistry in tissues with negative PAS staining [34].

Macrophages containing the *Tropheryma whipplei* organism were aggregated in the submucosa of our patient’s small bowel rather than their usual location in the lamina propria of the duodenal and jejunal villi. Such a submucosal small bowel localization of the organism has been reported previously in one case and was thought to be related to prior antibiotic therapy [35]. Our patient had not taken antibiotics prior to his small bowel biopsy; however, it is difficult to estimate the impact that his prior immunosuppressive therapy might have had on his enteric biopsy findings. This observation reinforces the need to obtain biopsies that include adequate submucosal tissue.

Our patient’s diagnosis of Whipple’s disease was delayed because of his atypical presentation with constrictive pericarditis. This case highlights that pericarditis may occur as a predominant manifestation of this disease, even without concomitant gastrointestinal symptoms. The same has been shown for endocarditis [30]. His pericardial tissue was not stained specifically for the organism at the time of the original biopsy. The organisms were later identified when the tissue was retrieved and specifically stained for *Tropheryma whipplei*. This sequence of events is similar to that described by Makol et al. [9]. Ongoing immunosuppressive treatment during this period of diagnostic uncertainty may have contributed to the progression of his disease with the development of severe fibrosing pleuritis [36-38].

The therapeutic experience related to Whipple’s constrictive pericarditis is limited. As seen in Table 1, pericardiectomy followed by antibiotic therapy is an approach with good reported outcomes. Our patient was treated medically without pericardiectomy. This decision was predicated on the known morbidity of pericardial stripping, especially in a patient with advanced cardiac cachexia, and the ability to manage his right-sided heart failure medically.

## Conclusion

Pleuropericardial involvement is a common pathologic finding in patients with Whipple’s disease, but rarely causes clinical symptoms. We report an elderly man who presented with constrictive pericarditis and later developed dense pleural fibrosis requiring decortication. A diagnosis of Whipple’s disease was delayed by his atypical presentation. Constrictive pericarditis may occur as a predominant manifestation of Whipple’s disease, even in the absence of gastrointestinal symptoms. The diagnosis may be facilitated by PCR analysis of a peripheral blood sample.

## Consent

Consent for publication was obtained from patient’s next of kin and is available for review upon request.

## Competing interests

The authors declare that they have no competing interests.

## Authors’ contributions

Dr. GS conceived the paper, performed a literature review, drafted the manuscript and revised it critically for important intellectual content. Dr. AB assisted in drafting the manuscript, revised it critically for important intellectual content, and gave final approval of the version to be published. Dr. MM revised the manuscript for important intellectual content. Dr. PI revised the manuscript for important intellectual content and provided the histopathology pictures. Dr. SK revised the manuscript for important intellectual content. All authors read and approved the final manuscript.

## Authors’ information

Dr. George Stojan was supported by NIH Grant T32 AR048522.

Dr. Peter Illei has been a consultant to Leica, a manufacturer of laboratory equipment that was used in this case.

## Pre-publication history

The pre-publication history for this paper can be accessed here:

http://www.biomedcentral.com/1471-2334/13/579/prepub
